# A 16-Month Community-Based Intervention to Increase Aspirin Use for Primary Prevention of Cardiovascular Disease

**DOI:** 10.5888/pcd11.130378

**Published:** 2014-05-15

**Authors:** Niki C. Oldenburg, Sue Duval, Russell V. Luepker, John R. Finnegan, Heather LaMarre, Kevin A. Peterson, Nicole D. Zantek, Ginny Jacobs, Robert J. Straka, Karen H. Miller, Alan T. Hirsch

**Affiliations:** Author Affiliations: Sue Duval, Russell V. Luepker, John R. Finnegan, Heather LaMarre, Kevin A. Peterson, Nicole D. Zantek, Ginny Jacobs, Robert J. Straka, Karen H. Miller, Alan T. Hirsch, University of Minnesota, Minneapolis, Minnesota.

## Abstract

**Introduction:**

Cardiovascular diseases are the leading causes of disability and death in the United States. Primary prevention of these events may be achieved through aspirin use. The ability of a community-based intervention to increase aspirin use has not been evaluated. The objective of this study was to evaluate an educational intervention implemented to increase aspirin use for primary prevention of cardiovascular disease in a small city in Minnesota.

**Methods:**

A community-based intervention was implemented during 16 months in a medium-sized community in Minnesota. Messages for aspirin use were disseminated to individuals, health care professionals, and the general population. Independent cross-sectional samples of residents (men aged 45–79, women aged 55–79) were surveyed by telephone to identify candidates for primary prevention aspirin use, examine their characteristics, and determine regular aspirin use at baseline and after the campaign at 4 months and 16 months.

**Results:**

In primary prevention candidates, regular aspirin use rates increased from 36% at baseline to 54% at 4 months (odds ratio = 2.05; 95% confidence interval, 1.09–3.88); the increase was sustained at 52% at 16 months (odds ratio = 1.89; 95% confidence interval, 1.02–3.49). The difference in aspirin use rates at 4 months and 16 months was not significant (*P* = .77).

**Conclusion:**

Aspirin use rates for primary prevention remain low. A combined public health and primary care approach can increase and sustain primary prevention aspirin use in a community setting.

## Introduction

Cardiovascular diseases (CVD), specifically acute myocardial infarction (AMI) and ischemic stroke, are the leading causes of disability and death in the United States (1). Although significant progress in the prevention, detection, and treatment of these diseases has been made, additional reductions in CVD events would provide a major societal benefit (2). Primary prevention strategies offer the most effective and cost-effective means to reduce CVD morbidity and mortality. One evidence-based primary prevention approach is the use of low-dose aspirin.

In 1989, the Physicians’ Health Study showed the benefit of low-dose aspirin in preventing AMI among men (3). Similar results for ischemic stroke were confirmed in the Women’s Health Study (4). These and other prospective randomized, placebo-controlled trials provide a high degree of confidence in the efficacy and safety of this approach (5,6). A meta-analysis of primary prevention data showed that aspirin use among healthy adults achieved a significant 12% relative-risk reduction in nonfatal CVD events (7). These data led the US Preventive Services Task Force (USPSTF) in 2009 to provide an A-grade recommendation for use of low-dose aspirin for primary prevention in men aged 45 to 79 years and women aged 55 to 79 years, where the potential benefit due to a reduction in AMI for men and stroke for women outweighs the potential harm due to an increase in gastrointestinal hemorrhage (8). This recommendation is recognized by the Centers for Disease Control and Prevention (CDC) *Healthy People 2020* objectives as an important goal (9) and is supported by the American Heart Association (AHA) Guidelines for Primary Prevention of Cardiovascular Disease and Stroke (10,11), the Million Hearts initiative of the US Department of Health and Human Services, the Centers for Medicare and Medicaid Services (12), and studies published since the recommendation was made (13–15).

Despite promotion of these guidelines, studies on primary prevention aspirin use in various populations have demonstrated low rates of use ranging between 10% and 40% (16–20). In Minnesota, rates of aspirin use are low (20%–35%) among aspirin candidates in the Minneapolis–St. Paul metropolitan area (21). Increasing rates of aspirin use for primary prevention has the potential to reduce rates of first AMI in men and first stroke in women, but the best method for increasing population-based aspirin use among those who would likely benefit from this therapy has yet to be determined.

Community-based interventions offer the greatest potential for population-level reductions in CVD morbidity and mortality (22), but they have been inconsistent in achieving clinically relevant improvements in CVD risk factors and a net change in CVD risk. Most community risk-reduction studies were designed to address multiple modifiable CVD risk factors, such as tobacco use, diet, exercise, hypertension, and cholesterol (23–25). More recent evidence shows community-wide educational campaigns to be effective in addressing CVD risk factors such as exercise, tobacco use, alcohol use, and diabetes, and shows primary care and patient approaches to be effective in addressing high blood pressure and high cholesterol (26). No interventions, to our knowledge, have been developed to reduce CVD morbidity and mortality through the promotion of a single cardiovascular pharmacologic approach, such as aspirin use.

We developed a community-based intervention, involving public health and primary care approaches, to increase primary prevention aspirin use among the USPSTF-recommended population in a medium-sized community in Minnesota. This study examines the characteristics of candidates for primary prevention aspirin use, aspirin use among candidates, and the impact of the intervention on aspirin use.

## Methods

### Community setting

Hibbing, Minnesota, was selected for the community-based intervention. The city (www.hibbing.mn.us) has a population of approximately 16,000, is predominantly white, has a median age of 43 years, has a median household annual income of $38,000, and 28% of its residents are college graduates (27). It is representative of other Minnesota municipalities but may be more racially homogenous than other small cities outside the Midwest. Three health systems offer primary care to this community.

### Community-based intervention

We conducted focus groups for the general public and for primary care physicians to inform the development of the intervention and then used a 3-tiered approach for message dissemination. First, as a one-on-one approach, we developed self-assessment tools to provide individuals with a quick means to self-identify as a primary prevention aspirin candidate who should discuss aspirin use with a health care professional. These tools were distributed via primary care clinics, pharmacies, work sites, and public and private organizations. Individuals were also identified in primary care clinics as aspirin-eligible candidates and were managed directly by medical staff and physicians. Second, a group-level intervention was promoted among primary care health professionals who were provided with continuing medical education (CME)-certified training sessions (1.0 credit hour for physicians, physician assistants, and nurses) and clinic tools to identify candidates and facilitate aspirin prescriptions. Fifty-three percent (16/30) of the primary care physicians and nurse practitioners and 66% (56/85) of the primary care medical staff (registered nurses, licensed practical nurses, and medical assistants) across the 3 health systems completed the CME-certified training sessions. Finally, a community-wide intervention included a mass media campaign that directed individuals in the USPSTF age- and sex-recommended candidacy range to consult with their health professionals to complete an individual risk assessment. The intervention also created partnerships with community organizations to address use of aspirin at the population level for primary prevention.

Primary prevention aspirin messages were disseminated in 2 sequential waves from March 2012 through June 2012 and from January 2013 through April 2013 through various media and public outlets, including daily print advertisements, 30 radio spots per week, billboards, online advertisements, and a website. A formal commitment to program goals through a signed memorandum of understanding was obtained from the 3 health systems. Program staff completed all CME-certified training sessions in the primary care clinics in the health systems by April 2012, and aspirin-candidacy clinic tools were used through April 2013.

### Evaluation design and assessment

Independent cross-sectional samples of community residents were surveyed at baseline, at 4 months (after the first media campaign), and at 16 months (after the second media campaign). The survey sampling frame included residents in households with landline telephones located in the city zip code area. Study contact consisted of 1 informational letter followed by as many as 10 call attempts per household. Trained interviewers from the Minnesota Center for Survey Research (http://oms.umn.edu/mcsr/) screened telephone respondents and administered a 10-minute telephone survey to men aged 45 to 79 years and women aged 55 to 79 years. For the baseline and 4-month surveys, we administered the survey to both primary and secondary prevention candidates. Because our study focused on primary prevention aspirin use, we did not survey secondary prevention candidates for the 16-month survey; we surveyed only primary prevention candidates. We oversampled women in the 4-month and 16-month surveys to achieve a balanced sex ratio. Response rate was defined as the percentage of completed interviews among telephone-to-person contacts made, excluding sex- or age-ineligible respondents. Data collection methods precluded a precise estimate of individual Framingham risk scores (8).

The telephone survey included questions designed to evaluate sociodemographic characteristics, history of CVD and atherosclerosis risk factors, aspirin use, psychosocial characteristics, and exposure to CVD health messages. Consistent with AHA and USPSTF definitions (8,10), candidates for aspirin use for primary prevention were defined as individuals who did not have a self-reported history of CVD, including heart attack, ischemic stroke, atherosclerotic lower extremity peripheral artery disease (PAD), or any heart-related, stroke-related, or PAD-related revascularization procedure. Aspirin use was examined by using the following question: “How often do you take aspirin?” Regular aspirin use was defined as use daily or every other day. Nonregular aspirin use was defined as once per week or less. No minimum duration of aspirin use was assessed in defining regular use. Individuals in the baseline sample were also invited to have their blood drawn to validate self-reported aspirin use through a serum thromboxane B_2_ (STxB_2_) measurement (Thromboxane B_2_ EIA Kit, Cayman Chemical, Ann Arbor, Michigan). Self-reported aspirin use from this survey was validated in a related study to be an accurate representation of actual use via measurement of STxB_2_ levels (28). Approval for the study was obtained from the University of Minnesota institutional review board.

### Analysis

Most analyses were limited to candidates for aspirin use. Regular aspirin use was compared across the surveys. Unadjusted odds ratios (ORs) with 95% confidence intervals (CIs) were calculated by using Stata version 12 (StataCorp LP, College Station, Texas). Logistic regression analyses found that sociodemographic variables did not confound associations, so we calculated unadjusted ORs only. We used χ^2^ tests for categorical variables and *t* tests and analysis of variance for continuous variables.

## Results

Telephone survey response rates were 56% at baseline, 51% at 4 months, and 40% at 16 months. The sociodemographic distribution of the respondents was similar across the surveys except for the oversampling of women at 4 months and 16 months (Table 1). The sociodemographic distribution was also representative of the community population.

**Table 1:** Sociodemographic Characteristics of Primary Prevention Candidates at Baseline, 4-Month, and 16-Month Surveys in a Small City in Minnesota, 2012–2013^a^

Sociodemographic characteristic	Baseline (N = 74)	4-Month (N = 85)	16-Month (N = 102)	*P* Value^b^
**Sex, no of respondents**	74	85	102	NA
Female	24 (32)	42 (49)	53 (52)	NA
Male	50 (68)	43 (51)	49 (48)	
**Age, mean (SD), y**	62 (9)	64 (8)	64 (8)	.28
**Age group, no. of respondents**	74	84	101	NA
45–54 y (men only)	17 (23)	8 (10)	12 (12)	.06
55–64 y	30 (41)	39 (46)	36 (36)	
65–74 y	17 (23)	25 (30)	42 (42)	
75–79 y	10 (14)	12 (14)	11 (11)	
**Marital status, no. of respondents**	74	83	102	NA
Married	51 (69)	60 (72)	71 (70)	.86
Single	6 (8)	5 (6)	4 (4)	
Divorced/separated	7 (9)	10 (12)	14 (14)	
Widowed	10 (14)	8 (10)	13 (13)	
**Education, no. of respondents**	74	81	102	NA
High school or less	25 (34)	25 (31)	25 (25)	.32
Some postsecondary	30 (41)	26 (32)	45 (44)	
College graduate	19 (26)	30 (37)	32 (31)	
**Annual household income, no. respondents**	66	79	91	NA
Below $30,000	21 (32)	24 (30)	26 (29)	.96
$30,000–$60,000	20 (30)	26 (33)	33 (36)	
Above $60,000	25 (38)	29 (37)	32 (35)	

Abbreviations: NA, not applicable, SD, standard deviation.

a Values are number (percentage) unless otherwise indicated; categories may not sum to total because of missing data; women were oversampled in 4-month and 16-month surveys.

b χ^2^ test for categorical variables and one-way analysis of variance for continuous variables; *P* values are for differences across the 3 surveys.

Cardiovascular comorbidities and risk factors were similar across survey samples; approximately 11% reported having a heart attack, 4% a stroke, and 10% PAD. Approximately 19% reported having diabetes, 54% high cholesterol, and 47% high blood pressure; 13% were current smokers. Approximately two-thirds of the baseline and 4-month samples were primary prevention candidates. The distribution of age, sex, and risk factors suggest we identified an initially appropriate candidate pool for primary prevention through population-based measures, despite lack of Framingham risk scores.

Of the 103 people in the baseline sample, 54 (52%) agreed to participate in a substudy to adjudicate the accuracy of self-reported aspirin use. Of these, 31 reported aspirin use, and 29 (94%) had their aspirin use confirmed by the STxB_2_ measurement. 

The demographic and socioeconomic distribution of the aspirin use candidates was similar at baseline, 4 months, and 16 months (Table 1). Among the 3 samples, the mean age ranged from 62 to 64 years, and approximately two-thirds were married. More than 90% of the primary prevention candidates were white, and more than 94% had health insurance coverage, reflecting the demographic characteristics of the community population.

The associations between the sociodemographic subgroups and aspirin use were also similar across the primary prevention samples (Table 2). Increasing age was significantly associated with higher rates of aspirin use in all 3 samples; primary prevention candidates who used aspirin regularly were more likely to be older (aged 65–79) than younger (aged 45–64). Sex and education were not associated with aspirin use in any of the 3 samples. Income was significantly associated with aspirin use but only in the 4-month survey; regular aspirin users were more likely to have a low annual household income (<$30,000) than an income of $30,000 to $60,000 or more than $60,000. In addition, marital status was significantly associated with aspirin use but only in the 16-month survey.

**Table 2:** Prevalence of Regular Aspirin Use^a^ in Sociodemographic Subgroups Among Primary Prevention Candidates at Baseline, 4-Month, and 16-Month Surveys in a Small City in Minnesota, 2012–2013^b^

Subgroup	Baseline (N = 74)		4-Month (N = 85)		16-Month^c^ (N = 102)	
	Regular Aspirin Use (n = 27)	*P* Value^d^	Regular Aspirin Use (n = 46)	*P* Value^d^	Regular Aspirin Use (n = 52)	*P* Value^d^
**Sex**						
Female	10/24 (42)	.52	22/42 (52)	.75	32/53 (60)	.08
Male	17/50 (34)		24/43 (56)		20/47 (43)	
**Age group, y**						
45–54 (men only)	5/17 (29)	.07	3/8 (38)	.005	2/12 (17)	.05
55–64	7/30 (23)		16/39 (41)		18/34 (53)	
65–74	9/17 (53)		21/25 (84)		26/42 (62)	
75–79	6/10 (60)		6/12 (50)		6/11 (55)	
**Marital status**						
Married	16/51 (31)	.11	32/60 (53)	.60	35/69 (51)	.05
Single	1/6 (17)		2/5 (40)		0/4 (0)	
Divorced/separated	5/7 (71)		5/10 (50)		7/14 (50)	
Widowed	5/10 (50)		6/8 (75)		10/13 (77)	
**Education**						
High-school or less	12/25 (48)	.18	14/25 (56)	.75	11/25 (44)	.43
Some postsecondary	11/30 (37)		13/26 (50)		26/44 (59)	
College graduate	4/19 (21)		18/30 (60)		15/31 (48)	
**Annual household income**						
Below $30,000	9/21 (43)	.09	18/24 (75)	.03	13/26 (50)	.38
$30,000–$60,000	10/20 (50)		11/26 (42)		19/33 (58)	
Above $60,000	5/25 (20)		12/29 (41)		12/30 (40)	

a Regular aspirin use was defined as use daily or every other day.

b Values are numerator/denominator (percentage) unless otherwise indicated; categories may not sum to total because of missing data.

c For the 16-month survey, regular aspirin use could be determined for 100 of the 102 primary prevention respondents.

d χ^2^ test.

At baseline, 36% of the primary prevention candidates reported regularly using aspirin; at 4 months, the percentage was 54%. We found a 2-fold difference in regular aspirin use between the baseline and 4-month survey (OR = 2.05; 95% CI, 1.09–3.88) and between the baseline and 16-month survey (OR = 1.89; 95% CI, 1.02–3.49). Primary prevention aspirin use rates were sustained at 16 months (54% at 4 months, 52% at 16 months; *P* = .77).

At 4 months, 46% of primary prevention candidates and at 16 months, 63% of candidates indicated they had seen or heard the program messages in their community or workplace. When asked to identify their reasons for initiating aspirin use, most primary prevention candidates who took aspirin regularly to prevent a heart attack or stroke did so in response to a recommendation by their health care provider (68% at 4 months, 71% at 16 months); the next most common reason was response to media advertisements (66% at 4 months, 46% at 16 months). Fewer than 30% of candidates in both surveys reported initiating aspirin use in response to a friend or family recommendation, a close personal experience with someone who suffered a heart attack or stroke, or community information.

Aspirin use discussions initiated by health care providers also increased among primary prevention candidates, but this increase was not significant (12% at baseline; 19% at 4 months, 22% at 16 months). Regular aspirin users were significantly more likely than nonregular aspirin users to have had aspirin discussions with their health care provider at 4 months (OR = 4.62; 95% CI, 1.62–13.14) but not at 16 months (OR = 1.68; 95% CI, 0.69–4.10).

Regular and nonregular aspirin users differed significantly from each other on several measures of aspirin-related attitudes, perception of CVD risk, and social norms at 16 months (Figure). Regular aspirin users believed more strongly than nonregular aspirin users that aspirin use can help prevent a heart attack or stroke (*P* < .001) and that daily aspirin use is safe (*P* = .001) and effective (*P* = .04). The personal perception of CVD risk was similar among regular and nonregular aspirin users; both groups believed their chances of having a heart attack or stroke were low. The social norms among both groups differed significantly. Regular aspirin users believed more strongly that people similar to them take daily aspirin (*P* < .001) and that people close to them recommend they take aspirin (*P* < .001).

**Figure:**
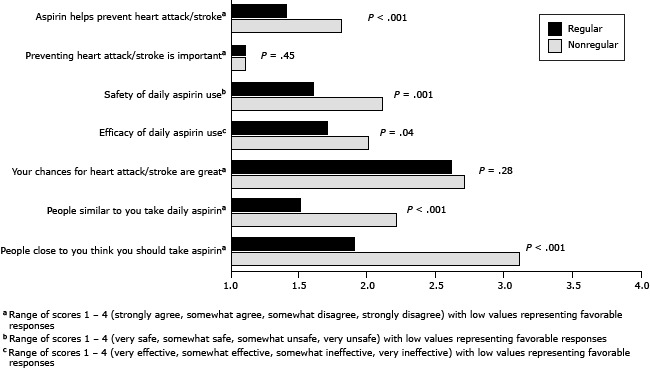
Differences in aspirin-related attitudes, perception of cardiovascular risk, and social norms between regular and nonregular aspirin users at 16 months. Regular aspirin use was defined as use daily or every other day. Nonregular aspirin use was defined as once per week or less. **Table:** 

Question	Type of Aspirin User	Mean Score	*P* Value
Aspirin helps prevent heart attack/stroke^a^	Nonregular use	1.8	<.001
Aspirin helps prevent heart attack/stroke^a^	Regular use	1.4	
Preventing heart attack/stroke is important^a^	Nonregular use	1.1	.45
Preventing heart attack/stroke is important^a^	Regular use	1.1	
Safety of daily aspirin use^b^	Nonregular use	2.1	.001
Safety of daily aspirin use^b^	Regular use	1.6	
Efficacy of daily aspirin use^c^	Nonregular use	2.0	.04
Efficacy of daily aspirin use^c^	Regular use	1.7	
Your chances for heart attack/stroke are great^a^	Nonregular use	2.7	.28
Your chances for heart attack/stroke are great^a^	Regular use	2.6	
People similar to you take daily aspirin^a^	Nonregular use	2.2	<.001
People similar to you take daily aspirin^a^	Regular use	1.5	
People close to you think you should take aspirin^a^	Nonregular use	3.1	<.001
People close to you think you should take aspirin^a^	Regular use	1.9	

^a^ Range of scores is 1 to 4 (strongly agree, somewhat agree, somewhat disagree, strongly agree), with low values representing favorable responses.

^b^ Range of scores is 1 to 4 (very safe, somewhat safe, somewhat unsafe, very unsafe), with low values representing favorable responses.

^c^ Range of scores 1 to 4 (very effective, somewhat effective, somewhat ineffective, very ineffective), with low values representing favorable responses.

## Discussion

During the past 25 years, the evidence base has expanded to demonstrate that aspirin use can prevent a first AMI in men and first ischemic stroke in women. Nevertheless, there is a paucity of research on how a community-based intervention might effectively encourage aspirin use for primary prevention to achieve the promised efficacy (26). Representative estimates from national (16,17) and state (18–21) population-based studies show aspirin use rates ranging from 10% to 40% among adults who are likely to benefit from this therapy (16–21). Our study showed a self-reported rate of aspirin use of 36% at baseline, which is comparable to the historical rate (36%), but which increased to 54% after 4 months and was sustained at 52% at 16 months. This increase is greater than that observed during past temporal trends.

Studies examining the association between sex and aspirin use (16–20) have shown inconsistent findings. Our study found no differences in aspirin use between men and women and no differences between regular and nonregular aspirin users in educational level. The homogeneity of our study participants in race/ethnicity and health insurance coverage prevented a meaningful evaluation of these factors, which are predictors of regular aspirin use and modify use of other prevention-directed medications.

Similar to regular aspirin users in other studies (16–20), aspirin users in our study were more likely to be older (aged 65–79). Because we included only individuals who met the USPSTF age and sex recommendations for primary prevention aspirin use, our study suggests that interventions may provide the greatest benefit for those aged 45 to 64, who are at higher risk of CVD events but who may not consider themselves to be at risk. Furthermore, regular aspirin users had a more favorable attitude toward aspirin use, and as in another study (29), they were more likely to perceive aspirin use as a common behavior in their social network. This latter finding supports the importance of influencing social networks to achieve aspirin use.

Community-based interventions offer an important means to increase population-based aspirin use for primary prevention. Studies on community-based interventions have demonstrated variable success in reducing CVD risk factors, morbidity, and mortality (24–26,30–32). Reasons for limited success may relate to the complexity of simultaneously targeting multiple CVD risk factors and multiple behavioral changes. In contrast, focusing on 1 message and 1 behavioral change to increase primary prevention aspirin use, as our study did, may be more manageable. Sequential 1-message interventions may be less overwhelming to target populations than multiple-message interventions.

Our study suggests that CVD interventions benefit from the involvement of both health care and non-health–care public and private community partners as either intervention facilitators or conduits of CVD prevention messages, as found previously (23). Media campaigns complement primary care aspirin initiatives by encouraging the public to initiate aspirin-related discussions with their health care providers. Health care professionals then play a strong role in helping individuals decide to use aspirin as a primary prevention measure (16). In line with a recent Institute of Medicine brief (33), maximal synergy and impact can be achieved by the sharing of information, resources, expertise, and credit across the primary care and public health communities.

Our study has several limitations. The evaluation provided a pre- and post-intervention design without a nonintervention comparison group. This design cannot fully account for temporal trends in societal aspirin use; nonetheless, the magnitude of change associated with the intervention far exceeds any temporal trends. Although CVD risk and aspirin use in our study may be subject to self-report bias, this bias is likely minimal, according to the work that adjudicated self-reported aspirin use via STxB_2 _measurement. This intervention was not designed, at this initial community dissemination stage, to evaluate the potential risk that could be incurred if inappropriate aspirin use were promoted. Individual 10-year CVD risk profiles based upon the Framingham Heart Study were not calculated for respondents, because we could not collect the physiologic data required to perform the calculation. Instead, candidacy for primary prevention aspirin use was determined by using the 2009 USPSTF age and sex recommendations, which are based on the Framingham risk scores. We did not examine contraindications to aspirin use, such as aspirin allergy, history of gastrointestinal bleeding, and the use of other antithrombotic or anti-inflammatory medications. Thus, it is not known whether regular aspirin users included individuals who should not be taking aspirin because of existing contraindications.

Rates of aspirin use for the primary prevention of CVD remain relatively low. Increasing appropriate aspirin use among a high-risk target population per USPSTF guidelines could reduce first AMI events in men and first ischemic stroke events in women and likely achieve these benefits cost-effectively. A combined public health and primary care approach, through the delivery of a community-based intervention, may offer an effective and relatively rapid means to increase and sustain aspirin use rates for primary prevention.
